# Evaluating a drink-counting and a breathalyzer-coupled app for monitoring alcohol use: A comparison with timeline followback and peth biomarker

**DOI:** 10.1016/j.abrep.2025.100643

**Published:** 2025-11-14

**Authors:** Josefine Östh, Andreas Lundin, Peter Wennberg, Sven Andréasson, Anna-Karin Danielsson

**Affiliations:** a Department of Global Public Health, Karolinska Institutet, Solnavägen 1E, 113 65 Stockholm, Sweden; b Centre for Epidemiology and Community Health, Stockholm Region, Solnavägen 1E, 113 65 Stockholm, Sweden; c Department of Public Health Sciences, Stockholm University, Albanovägen 12, 10690 Stockholm, Sweden; d Department of Psychology, Inland Norway University of Applied Sciences, Vormstuguvegen 2, 2624 Lillehammer, Norway

**Keywords:** Alcohol dependence, Heavy drinking, mHealth, PEth, Timeline Followback

## Abstract

**Aim:**

Accurately measuring alcohol consumption remains a challenge. This study aimed to evaluate two app-based methods, one using drink-count logging and one using a breathalyzer, by comparing them to retrospective self-report (Timeline Followback, TLFB) and a biomarker of alcohol use (Phosphatidylethanol, PEth).

**Methods:**

Data were acquired from a randomized controlled trial involving alcohol-dependent adults (n = 110). Standard drinks, drinking days, and heavy drinking days reported via the drink-counting app or breathalyzer, were compared with TLFB data over the 12-week period using Lin’s concordance correlation coefficient (CCC). Correlation with PEth was assessed only at the 12-week mark, using Spearman’s rank correlation coefficient (rho) and Receiver Operating Characteristic (ROC) curves, reporting the area under the curve (AUC).

**Results:**

Compared to app-based methods, TLFB consistently identified more drinking days and heavy drinking days. However, the drink-counting app’s estimates were still relatively close to TLFB and demonstrated strong agreement for drinking days across the different time intervals (CCC = 0.71–0.86). The drink-counting app also showed a strong correlation with PEth values for standard drinks and drinking days (rho = 0.74–0.78). In contrast, breathalyzer data generally showed weak agreement with both TLFB and PEth.

**Conclusions:**

Although TLFB yielded more drinking and heavy drinking days, the drink-counting app showed strong agreement with TLFB and correlated closely with PEth levels, indicating good validity. In contrast, breathalyzer data showed weaker agreement, likely due to lower usage during drinking episodes. These findings suggest that drink-counting apps could provide a reliable tool for monitoring alcohol use, offering advantages over both retrospective reports and breathalyzer measures.

## Highlights

• Alcohol use reported via app and breathalyzer was compared with traditional reports.
• App and breathalyzer reports were also compared with an objective biomarker.
• The app showed strong agreement with retrospective self-reports and objective data.
• Breathalyzer data weakly aligned with retrospective and objective reports.
• The app appears valid for tracking alcohol use, whereas the breathalyzer does not.

## 1 Introduction

The use of mobile devices, such as smartphones or tablets, to support and deliver health-related services, information, and interventions in healthcare (commonly referred to as mobile health or mHealth) is increasingly recognized as a valuable tool in the prevention and management of various diseases (World Health Organization, 2018). Among these, smartphone applications (apps) offer particularly promising opportunities for health interventions due to their flexibility, personalization, and specific functions—such as automatic reminders and their ability to integrate with external devices like heart rate monitors, blood sugar monitors, and breathalyzers (Businelle et al., 2024, Siegler et al., 2021). These features make apps especially suitable for supporting and studying behavioral change interventions.

While mHealth tools for alcohol monitoring are growing rapidly (Johansson et al., 2024), standard assessment of alcohol consumption still primarily relies on retrospective recall, most commonly the Timeline Followback (TLFB) method (Sobell & Sobell, 1992). However, retrospective self-reports are vulnerable to recall errors and social desirability bias, especially among individuals with alcohol dependence (Del Boca & Darkes, 2003). For example, accuracy can be affected by the mode of administration and complexity of a questionnaire, social desirability, and personal characteristics like age, sex, dependence severity, recovery stage, and the ability to recall events (Del Boca & Darkes, 2003). Relatedly, studies suggest that self-reported alcohol consumption is significantly underreported (de Bejczy et al., 2022, Guttormsson, 2020).

Ecological momentary assessment (EMA) via smartphone apps may mitigate these limitations by collecting consumption data repeatedly and immediately in real-life settings (Shiffman et al., 2008). Compared to retrospective assessments of self-reported alcohol consumption, EMA may reduce recall bias and provide better insight into consumption patterns (Beckjord & Shiffman, 2014). Moreover, integrating biomarkers such as phosphatidyl ethanol (PEth) may further improve precision by providing objective evidence of alcohol use over the preceding weeks (de Bejczy et al., 2022), verifying self-reported data and reducing bias (Perilli et al., 2023).

Previous studies comparing app-based EMA and conventional methods in sub- and non-clinical samples have shown mixed results. Dulin et al. (2017) found high agreement between TLFB and daily app reports over 6 weeks, though agreement declined over time. In contrast, two studies reported inconsistencies between TLFB reports and momentary or daily app-based methods over two and four weeks (Freeman et al., 2023, Merrill et al., 2020). However, a recent study comparing app-based daily reports and TLFB with PEth-values over two weeks found consistent results (Finanger et al., 2024). Only one study has included objective breathalyzer data; Hämäläinen et al. (2020) found that alcohol-dependent individuals in aftercare underreported drinking on TLFB, compared to breathalyzer reports, especially when aiming for abstinence (Hämäläinen et al., 2020).

Most previous studies have compared app-based daily or real-time assessments with recall in the past few weeks (typically within 2–4 weeks), and few have included clinical samples. We utilized data from a recent randomized controlled trial (RCT) examining two mHealth tools (a drink-counting app and an app-coupled breathalyzer) in alcohol-dependent adults over a 12-week intervention (Östh et al., 2025). Participants were instructed to use these tools daily, and repeatedly during drinking occasions, and to complete the TLFB retrospectively at the 12-week assessment. In addition, blood samples were collected for PEth analysis at 12 weeks.

The aim of this study was to compare alcohol consumption reports from a drink-counting app and an app-connected breathalyzer with those obtained through the TLFB method, and the biomarker PEth, in a sample of alcohol-dependent adults.

### 1.1 Research questions and hypothesis

1. To what extent does alcohol consumption, as continuously reported in the drink-counting app and breathalyzer, agree with consumption reported retrospectively using the TLFB method?
2. How do alcohol consumption patterns differ across these measurement methods?
3. How accurately do consumption reports from the drink-counting app and breathalyzer predict whether consumption is sporadic, moderate, or heavy, as determined by PEth?

We hypothesized that alcohol consumption would be reported as higher when assessed with the drink-counting app and breathalyzer than with TLFB, and that app reports would align more closely with PEth levels than with TLFB. We also expected stronger agreement between methods near the 12-week assessment, when TLFB recall was assumed to be most accurate.

## 2 Material and methods

### 2.1 Participants and procedure

Data were acquired from 110 out of the 162 alcohol-dependent adults who participated in a three-arm parallel RCT conducted between 15 September 2020 and 30 June 2023 at Riddargatan 1, an outpatient clinic within the Stockholm Centre for Dependency Disorders. The trial has been described in detail elsewhere (Danielsson et al., 2018, Östh et al., 2025) (trial registration: ISRCTN14515753).

In brief, alcohol-dependent adults (46 % female) seeking treatment were randomized to one of three groups:

1. TAU + drink-counting app (n = 52)
2. TAU + app-coupled breathalyzer (n = 58)
3. TAU only (n = 52)

TAU (treatment as usual) consisted of four sessions of clinician-led psychological treatment based on motivational interviewing and cognitive behavioral therapy, combined with pharmacotherapy when requested, over a 12-week period. All participants met the ICD-10 criteria for alcohol dependence and reported at least five heavy drinking days (HDDs) in the eight weeks prior to inclusion.

Participants were assessed by a study coordinator at baseline at 12 weeks (post-intervention), and at 26 weeks (follow-up), using paper Case Report Forms (CRFs). For the present study, only participants randomized to using one of the apps, and only data from the 12-week assessment were analyzed, as this period corresponded with the active treatment phase during which the drink-counting app and breathalyzer were used. The completion date of the 12-week assessment was designated as the intervention end date. The 12-week period (84 days) preceding this date was defined as the intervention period for each participant (Figure A, Supplementary file).

The results of the original study have formerly been reported, including heavy drinking days, weekly alcohol consumption and PEth as outcome measures (Östh et al., 2025). The present study reports comparisons of these data with the app-based reports, not previously reported.

### 2.2 Ethical considerations

This study was approved by the Regional Ethics Committee in Stockholm (ref no. 2018/174–31) and the Swedish Ethical Review Authority (ref no. 2021–01965). Each participant provided written informed consent, and all data were kept confidential using pseudonymized study identifiers.

### 2.3 Instant (app-based) measures

The drink-counting app was designed for direct and repeated prospective registration of standard drinks (12 g/drink). Participants were instructed to use the app daily to record their consumption, including zero-drinking days, and to log each drink immediately after consumption during drinking sessions.

The breathalyzer, connected to the smartphone app via Bluetooth, was used to measure breath alcohol concentration (BrAC). BrAC is expressed in grams/210 L of breath and is equivalent to blood alcohol concentration (grams/100 ml of blood) (Jones, 2019). Participants received three daily notifications to use the breathalyzer (morning, evening, and one intermittent prompt) and were also instructed to take BrAC measurements hourly during drinking sessions. The app enabled notifications, BrAC testing, and review of the results.

The output from both apps were accessible for the clinicians in two separate online portals, for use as a basis for discussion during treatment sessions. Supplementary file, B and C, for intervention details.

Adherence, defined as using the drink-counting app or breathalyzer on at least 80 % of days during the 12-week intervention, was 81 % for the drink-counting app and 69 % for the breathalyzer (Figure D in the Supplementary file) (Östh et al., 2025).

### 2.4 Retrospective subjective measures

Alcohol consumption in the preceding 12 weeks (84 days) was assessed by using the TLFB method (Sobell & Sobell, 1992), which is a structured interview that helps individuals retrospectively report their daily alcohol consumption over a specified period using a calendar and memory aids. Participants were asked to recall and report the number of standard alcoholic drinks they consumed on each individual day, starting with the day before the interview and moving backward, day by day, until they had accounted for their alcohol consumption over the past 12 weeks. Using app-based registrations was not permitted.

### 2.5 Retrospective objective measures

Phosphatidyl ethanol (PEth) is formed on red blood cells in the presence of ethanol. PEth 16:0/18:1 is the homolog of the PEth group of phospholipids commonly chosen for laboratory measurements. It is thus an alcohol-specific, direct biomarker that is accumulated upon repeated intake and has a slow elimination rate, thus reflecting the past few weeks’ consumption (Helander and Hansson, 2023, Perilli et al., 2023). A value below 0.05 μmol/liter indicates no or low consumption, while a value above 0.30 μmol/liter suggests regular and high alcohol intake, with prolonged heavy consumption potentially resulting in much higher values (Helander & Hansson, 2023). Participants were instructed to have the test done at a laboratory at least three working days prior to the scheduled follow-up assessment.

### 2.6 Statistical analysis

The analysis plan was prospectively registered at the Open Science Framework (osf.io/sw9hr). Deviations from the preregistered plan included omitting the AUDIT-C comparisons to focus the manuscript and excluding abstinent days, as drinking and heavy drinking days were already reported.

The number of drinking days (DD) and heavy drinking days (HDD) recorded by the drink-counting app and breathalyzer, respectively, were compared with data from the TLFB method. DD was defined as registering any standard drink or a BrAC level of > 0 g%. Consumption was assessed the past week, past two weeks, past four weeks, second month, third month, and the entire 12-week (84-day) period. These intervals were chosen based on the RCT design, which was aligned with the clinic’s routine treatment schedule.

HDD was defined as days consuming at least 4 standard drinks, with one drink containing 12 g of 100 % ethanol (Swedish National Board of Health and Welfare, 2023), or days with a BrAC-value of at least 0.06 g% (Fisher et al., 1987). For the breathalyzer group, we consistently used the highest daily registered BrAC (peak BrAC).

Agreement was assessed using Lin’s concordance correlation coefficient (CCC), which measures both precision and accuracy, and can accommodate data with different means or variances (Lin, 1989). This makes CCC particularly suitable for alcohol use data, which are often skewed or zero-inflated (Liu et al., 2019). The following cut-offs were applied: weak (<0.50), moderate (0.50–0.70), strong (0.71–0.90), and excellent (>0.90) (Mukaka, 2012, Nelson and Allen, 2019).

To examine how consumption differed across measurement methods, means, medians, and standard deviations of DD, and HDD in the drink-counting app and breathalyzer respectively were compared with TLFB.

Correlations between recorded alcohol consumption and PEth values were examined by comparing the number of standard drinks, DD and HDD logged in the two weeks surrounding the 12-week assessment. Spearman’s rank correlation coefficient was used due to the differing data types. Correlation strength was classified as negligible (<0.30), weak (0.30–0.49), moderate (0.50–0.70), strong (0.71–0.90), or very strong (>0.90) (Mukaka, 2012).

Furthermore, differences in accuracy were tested, by evaluating how well total number of standard drinks, DD, and HDD over the past two weeks in the drink-counting app and breathalyzer predicted low (<0.05 μmol/liter), moderate (<0.05–0.30 μmol/liter), or high (>0.30 μmol/liter) PEth-levels (Helander & Hansson, 2023). Receiver Operating Characteristic (ROC) curves were constructed, and the area under the curve (AUC) was reported, with higher values indicating better model performance (de Hond et al., 2022). PEth-samples taken more than two weeks before or after the 12-week assessment date were excluded (Helander et al., 2019).

A complete case analysis was conducted, including only participants randomized to either the drink-counting app or the breathalyzer (n = 110), with those missing any relevant data excluded. SAS software, version 9.4 of the SAS System for Windows. Copyright © 2020, SAS Institute Inc. was used for analyzing the data.

## 3 Results

### 3.1 Sample characteristics

The sample included 46 % females and had a mean age of 51 years (SD = 12) (Table 1). The majority (79 %) were employed, with a university education (68 %). Most participants (91 %) aimed to reduce their alcohol consumption rather than achieve total abstinence. On average, participants reported 13 heavy drinking days (HDD) in the past four weeks (SD = 8) and consumed 25 standard drinks in the past week (SD = 17).

**Table 1:** Baseline characteristics of the randomized controlled trial sample (n = 110).

**Baseline characteristics**	**TAU + drink-counting app (n = 52)**	**TAU + breathalyzer** **(n = 58)**	**All** **(n = 110)**	**p-value**
***Age***	***mean (SD)***	***mean (SD)***	***mean (SD)***	0.933^a^
male	49.93 (12.56)	50.91 (13.00)	50.47 (12.71)	
female	51.16 (12.26)	50.52 (11.26)	50.84 (11.65)	
***Sex***	***% (n)***	***% (n)***	***% (n)***	0.601^b^
male	51.9 (27)	56.9 (33)	54.5 (60)	
female	48.1 (25)	43.1 (25)	45.5 (50)	
***Income***	***% (n)***	***% (n)***	***% (n)***	0.668^c^
employment	83.7 (41)	75.0 (42)	79.1 (83)	
pension	10.2 (5)	10.7 (6)	10.5 (11)	
sickness benefit/activity compensation	2.0 (1)	0.0 (0)	0.95 (1)	
study grant	0.0 (0)	0.0 (0)	0.0 (0)	
un-employment benefit/severance grant	0.0 (0)	1.8 (1)	0.95 (1)	
social security contributions	0.0 (0)	0.0 (0)	0.0 (0)	
other/mix	4.1 (2)	12.5 (7)	8.56 (9)	
***Education***	***% (n)***	***% (n)***	***% (n)***	0.259^c^
less than elementary school	0.0 (0)	0.0 (0)	0.0 (0)	
elementary school	2.2 (1)	9.1 (5)	5.9 (6)	
upper-secondary school	28.2 (13)	20.0 (11)	23.8 (24)	
university	67.4 (31)	69.1 (38)	68.3 (69)	
other	2.2 (1)	1.8 (1)	1.98 (2)	
***Accomodation***	***% (n)***	***% (n)***	***% (n)***	0.023*^c^
no accomodation	0.0 (0)	0.0 (0)	0.0 (0)	
rental	13.0 (6)	28.6 (16)	21.6 (22)	
apartment	32.6 (15)	44.6 (25)	39.2 (40)	
villa/terraced house	47.8 (22)	26.8 (15)	36.3 (37)	
rented room	4.4 (2)	0.0 (0)	1.96 (2)	
student apartment	0.0 (0)	0.0 (0)	0.0 (0)	
other/mix	2.2 (1)	0.0 (0)	0.98 (1)	
***Marital status***	***% (n)***	***% (n)***	***% (n)***	0.751^c^
never been married	10.4 (5)	17.9 (10)	14.4 (15)	
married/partnership	45.8 (22)	41.1 (23)	43.3 (45)	
cohabiting	25.0 (12)	19.6 (11)	22.1 (23)	
divorced/separated	14.6 (7)	19.6 (11)	17.3 (18)	
widow/widower	2.1 (1)	1.8 (1)	1.9 (2)	
other/mix	2.1 (1)	0.0 (0)	0.96 (1)	
***Previous treatment***	***% (n)***	***% (n)***	***% (n)***	0.329^c^
no	68.8 (33)	66.1 (37)	67.3 (70)	
“Alcoholics Anonymous”	2.1 (1)	0.0 (0)	0.96 (1)	
“Länkarna”	6.2 (3)	16.1 (9)	11.5 (12)	
alcohol clinic	6.2 (3)	3.6 (2)	4.8 (5)	
psychiatric clinic	2.1 (1)	0.0 (0)	0.96 (1)	
primary care center	2.1 (1)	7.1 (4)	4.8 (5)	
social services	2.1 (1)	0.0 (0)	0.96 (1)	
treatment home	0.0 (0)	0.0 (0)	0.0 (0)	
other/mix	10.4 (5)	7.1 (4)	8.7 (9)	
***Treatment goal***	***% (n)***	***% (n)***	***% (n)***	0.922^c^
abstinence	6.0 (3)	7.1 (4)	6.6 (7)	
cut down	90.0 (45)	91.1 (51)	90.6 (96)	
other	4.0 (2)	1.8 (1)	2.8 (3)	
	***mean (SD)***	***mean (SD)***	***mean (SD)***	
***HDD past month***	12.85 (7.66)	13.91 (7.81)	13.41 (7.72)	0.481^d^
***Total drinks past week***	24.27 (15.22)	24.94 (18.49)	24.62 (16.95)	1.000^d^
***ICD-10 criteria fulfilled***	3.79 (0.87)	3.98 (0.93)	3.89 (0.90)	0.253^d^
***AUDIT-C score (total n = 102)***	8.22 (1.35)	8.82 (1.79)	8.55 (1.63)	0.057^d^
***SADD score (total n = 99)***	10.98 (4.28)	12.57 (6.06)	11.83 (5.34)	0.225^d^
***PEth value (total n = 108)***	0.60 (0.60)	0.73 (0.52)	0.66 (0.56)	0.071^d^

Table excluding those randomized to treatment as usual alone. HDD = heavy drinking days; ICD = International classification of diseases; AUDIT-C = alcohol use disorder identification test consumption; SADD = short alcohol dependence data questionnaire; PEth = phosphatidylethanol; TAU = treatment as usual; a = *t*-test; b = chi-2 test; c = Fisher’s exact test; d = Wilcoxon rank sum test; *p < 0.05.

### 3.2 Mean consumption across different methods

The mean DD and HDD were consistently higher when reported using the TLFB method compared to recordings from the drink-counting app or breathalyzer (Table 2).

**Table 2:** Descriptive statistics of the mean and median number of drinking days, and heavy drinking days assessed by Timeline Followback, a drink-counting app, and a breathalyzer at different time intervals during the 12-week intervention.

		**Past week**			**Past 2 weeks**			**Past 4 weeks**			**Week 5–8**			**Week 9–12**			**Past 12 weeks**		
**Drinking days (DD)**		**n**	**mean (SD)**	**median**	**n**	**mean (SD)**	**median**	**n**	**mean (SD)**	**median**	**n**	**mean (SD)**	**median**	**n**	**mean (SD)**	**median**	**n**	**mean (SD)**	**median**

	TLFB^1^	45	3.27 (2.08)	3.00	45	6.58 (4.00)	6.00	45	13.67 (7.84)	12.00	44	14.64 (7.86)	15.00	44	14.73 (7.94)	13.00	44	43.27 (22.51)	43.50
	Drink-counting app	37	2.54 (1.91)	2.00	37	5.35 (3.57)	6.00	37	11.16 (6.86)	11.00	41	12.59 (6.88)	12.00	41	12.78 (6.20)	11.00	34	35.15 (18.35)	32.00
		**n**	**mean (SD)**	**median**	**n**	**mean (SD)**	**median**	**n**	**mean (SD)**	**median**	**n**	**mean (SD)**	**median**	**n**	**mean (SD)**	**median**	**n**	**mean (SD)**	**median**
	TLFB^2^	55	3.09 (2.29)	3.00	55	5.91 (4.06)	6.00	55	11.95 (7.82)	11.00	54	12.22 (8.23)	11.50	54	11.69 (8.66)	10.50	54	35.56 (21.52)	32.50
Breathalyzer	38	1.58 (1.50)	1.00	37	3.08 (2.38)	3.00	35	5.77 (4.21)	6.00	48	7.85 (5.96)	7.00	34	8.71 (6.62)	8.00	21	22.33 (15.29)	19.00
**Heavy drinking days (HDD)^a^**		**n**	**mean (SD)**	**median**	**n**	**mean (SD)**	**median**	**n**	**mean (SD)**	**median**	**n**	**mean (SD)**	**median**	**n**	**mean (SD)**	**median**	**n**	**mean (SD)**	**median**
	TLFB^1^	45	1.82 (1.53)	2.00	45	3.60 (2.53)	4.00	45	7.89 (5.22)	8.00	44	8.55 (6.75)	8.00	44	9.57 (7.86)	8.00	44	26.14 (18.44)	24.00
	Drink-counting app	37	1.41 (1.34)	2.00	37	2.81 (2.15)	3.00	37	5.59 (4.01)	6.00	41	7.88 (5.17)	7.00	41	7.76 (5.45)	7.00	34	19.76 (12.17)	18.50
		**n**	**mean (SD)**	**median**	**n**	**mean (SD)**	**median**	**n**	**mean (SD)**	**median**	**n**	**mean (SD)**	**median**	**n**	**mean (SD)**	**median**	**n**	**mean (SD)**	**median**
	TLFB^2^	55	1.84 (1.89)	1.00	55	3.69 (3.35)	3.00	55	6.98 (5.76)	7.00	54	6.85 (6.67)	5.00	54	6.80 (6.56)	5.50	54	20.61 (16.72)	16.50
	Breathalyzer	38	0.11 (0.39)	0.00	37	0.30 (0.57)	0.00	35	0.66 (1.19)	0.00	48	1.06 (1.44)	0.00	34	0.62 (1.13)	0.00	21	1.90 (2.96)	1.00

a=Heavy drinking days defined as 0.06 g% BrAC or consuming at least 4 standard drinks (à 12 g ethanol) on one occasion.

TLFB = Timeline Followback.

1 = TLFB reported by drink-counting app users; 2 = TLFB reported by breathalyzer users.NB! “Past week” means the most proximal week from the 12-week follow-up, i.e., the 12th week of intervention. “Week 9–12” refers to the most distant time of recall but represents the first weeks of intervention.

### 3.3 App–TLFB agreement

For the number of DD, there was strong agreement between TLFB and the drink-counting app across time intervals. Agreement for HDD was moderate overall but reached strong levels for reports from the past week (Table 3).

**Table 3:** Correlations between the number of drinking days and heavy drinking days reported by a drink-counting app and breathalyzer respectively, and Timeline Followback at different time intervals during the 12-week intervention.

		**Past week**		**Past 2 weeks**		**Past 4 weeks**		**Week 5–8**		**Week 9–12**		**Past 12 weeks**	
		**n**	**CCC (95 % CI)**	**n**	**CCC (95 % CI)**	**n**	**CCC (95 % CI)**	**n**	**CCC (95 % CI)**	**n**	**CCC (95 % CI)**	**n**	**CCC (95 % CI)**
**Drinking days (DD)**	TLFB^1^ vs. drink-counting app	36	0.78 (0.60, 0.88)	36	0.78 (0.60, 0.88)	36	0.84 (0.71, 0.92)	39	0.82 (0.68, 0.90)	39	0.71 (0.51, 0.83)	32	0.86 (0.72, 0.93)
	TLFB^2^ vs. breathalyzer	38	0.38 (0.17, 0.56)	37	0.28 (0.08, 0.47)	35	0.26 (0.05, 0.45)	47	0.48 (0.28, 0.63)	32	0.73 (0.55, 0.84)	21	0.66 (0.42, 0.81)
**Heavy drinking days (HDD)^a^**	TLFB^1^ vs. drink-counting app	36	0.72 (0.51, 0.85)	36	0.58 (0.31, 0.76)	36	0.61 (0.39, 0.77)	39	0.63 (0.40, 0.79)	39	0.69 (0.50, 0.82)	32	0.67 (0.43, 0.82)
	TLFB^2^ vs. breathalyzer	38	0.07 (−0.01, 0.15)	37	0.03 (−0.03, 0.08)	35	0.03 (−0.03, 0.09)	47	0.09 (0.02, 0.17)	32	0.06 (−0.01, 0.12)	21	0.03 (−0.04, 0.10)

a=Heavy drinking days defined as 0.06 g% BrAC or consuming at least 4 standard drinks (à 12 g ethanol) on one occasion.

TLFB = Timeline Followback; CCC=Concordance Correlation Coefficient (<0.50 = weak, 0.50–0.70 = moderate, 0.71–0.90 = strong, >0.90 = excellent agreement).

1 = TLFB reported by drink-counting app users; 2 = TLFB reported by breathalyzer users. “Past week” refers the most proximal week from the 12-week follow-up, i.e., the 12th week of intervention. “Week 9–12” refers to the most distant time of recall but represents the first weeks of intervention.

Agreement between TLFB and the breathalyzer for DD was generally weak, although it improved during weeks 9–12 and across the full 12-week intervention period. In contrast, agreement for HDD remained consistently very weak throughout (Table 3).

### 3.4 App–PEth correlation

There was a strong positive correlation between PEth values and both total standard drinks and DD reported in the drink-counting app over the past two weeks, and a moderate correlation with HDD. In contrast, correlations between PEth and both DD and HDD recorded by the breathalyzer were negligible (Table 4).

**Table 4:** Correlations between phosphatidylethanol in blood (PEth) and total standard drinks, number of drinking days, and heavy drinking days in the past two weeks reported by the drink-counting app and by the breathalyzer.

**In the past 2 weeks:**		**n**	**rho^b^ (95 % CI)**
**Total standard drinks**	PEth vs. drink-counting app	20	0.78 (0.51, 0.91)
**Drinking days**	PEth vs. drink-counting app	25	0.74 (0.48, 0.88)
	PEth vs. breathalyzer	25	0.21 (−0.20, 0.56)
**Heavy drinking days^a^**	PEth vs. drink-counting app	25	0.58 (0.23, 0.79)
	PEth vs. breathalyzer	25	0.11 (−0.30, 0.48)

a=Heavy drinking days defined as 0.06 g% BrAC or consuming at least 4 standard drinks (à 12 g ethanol) on one occasion.

b=Spearman's correlation coefficient (<0.30 = negligible, 0.30–0.49 = weak, 0.50–0.70 = moderate, 0.71–0.90 = strong, >0.90 = very strong correlation).

PEth = phosphatidylethanol measured in the 2 weeks before or after the 12-week assessment.

**Fig. 1:**
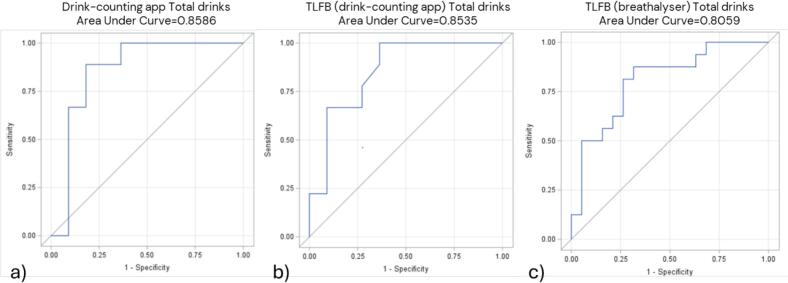
Receiver Operating Characteristic (ROC) curves for total standard drinks consumed in the two weeks before/after the 12-week assessment and having a high level of phosphatidylethanol (PEth) in blood. Total standard drinks reported by the drink-counting app (n = 20). Total standard drinks reported by Timeline Followback (TLFB) among users of the drink-counting app (n = 20). Total standard drinks reported by TLFB among users of the breathalyser (n = 35). A high PEth refers to a value > 0.30 μmol/liter; one standard drink equals 12 g of pure ethanol.

**Fig. 2:**
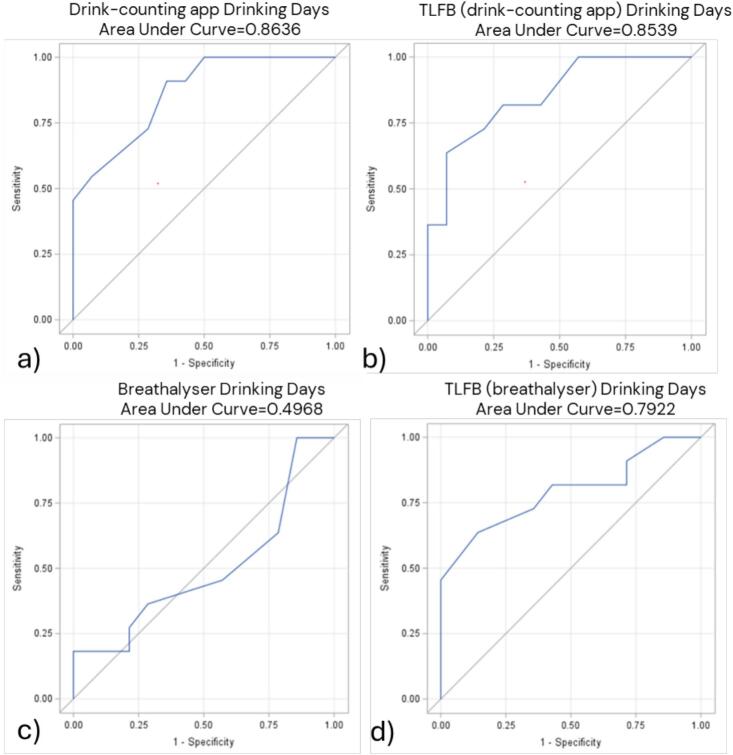
Receiver Operating Characteristic (ROC) curves for drinking days and having a high level of phosphatidylethanol (PEth) in blood two weeks before/after the 12-week assessment. Drinking days (DD) reported by the drink-counting app (n = 25). DD reported by Timeline Followback (TLFB) among users of the drink-counting app (n = 25). DD reported by the breathalyser (n = 25). DD reported by TLFB among users of the breathalyser (n = 25). A high PEth refers to a value > 0.30 μmol/liter.

**Fig. 3:**
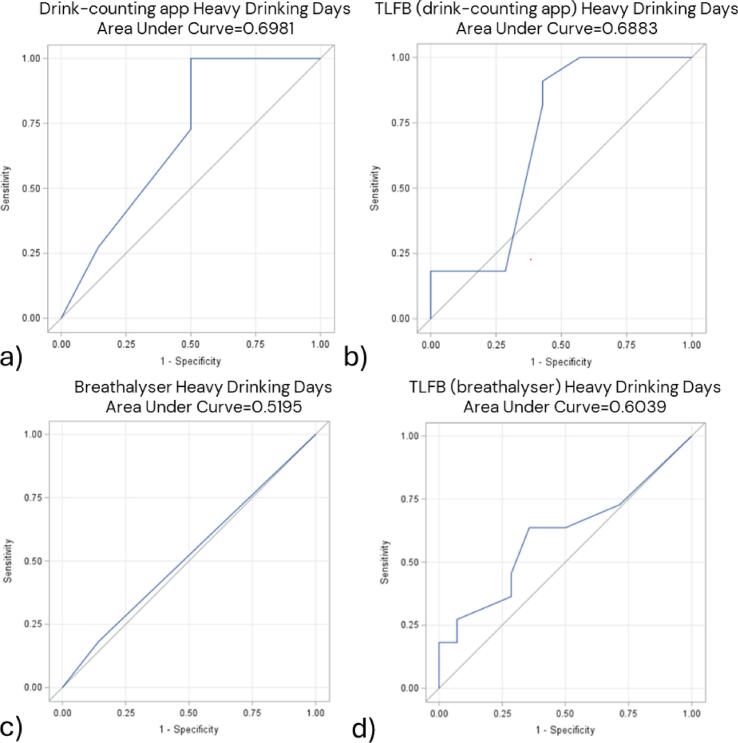
ROC-curves for heavy drinking days and having a high level of phosphatidylethanol (PEth) in blood two weeks before/after the 12-week assessment. Heavy drinking days (HDD) reported by the drink-counting app (n = 25). HDD reported by Timeline Followback (TLFB) among drink-counting app users (n = 25). HDD reported by the breathalyser (n = 25). HDD reported by Timeline Followback TLFB among users of the breathalyser (n = 25). A high PEth refers to a value > 0.30 μmol/liter.

ROC-curves based on the number of standard drinks and DD reported in the drink-counting app over the past two weeks resulted in an AUC of 0.86, indicating an 86 % probability of correctly classifying individuals with high PEth levels (Fig. 1, Fig. 2). The AUC for HDD reported in the app was lower, at 0.70 (Fig. 3). ROC curves based on DD and HDD recorded by the breathalyzer showed AUC values of 0.50 and 0.52, indicating a lack of meaningful diagnostic performance (Fig. 2, Fig. 3).

In all comparisons involving PEth, the drink-counting app outperformed TLFB, while the breathalyzer showed weaker performance than consumption reported by TLFB (Fig. 1, Fig. 2, Fig. 3).

## 4 Discussion

### 4.1 Main results

This study found mixed results regarding the agreement between prospective app-based reports of alcohol use and TLFB and PEth respectively. The level of agreement ranged from poor, or negligible, to excellent, depending on the type of measurement, time frame, and app used. For both the number of drinking days and heavy drinking days, agreement between TLFB and the drink-counting app ranged from moderate to excellent, while agreement with breathalyzer reports ranged from very weak to strong. Consistently, the agreement was poorest for heavy drinking days.

These results both align with and diverge from previous research. For example, Dulin et al (2017) found strong agreement with daily app-based reports, like our comparisons between TLFB and the drink-counting app. Although prior studies have shown that agreement tends to decline over time (Dulin et al., 2017), we found that agreement for the drink-counting app remained consistent over the study period. The high agreement between PEth and both TLFB and the drink-counting app reports is also consistent with the findings from a non-clinical sample in Norway (Finanger et al., 2024). In contrast, breathalyzer tests showed consistently weak agreement with TLFB and PEth.

The stronger agreement between TLFB and the drink-counting app may be explained by several factors. First, the drink-counting app was specifically designed to track standard drinks, which may have enhanced participants’ understanding of what constitutes a standard drink (Östh et al., 2023). This increased awareness could, in turn, have improved the accuracy of the TLFB reports.

Second, the drink-counting app and the breathalyzer were used in notably different ways during the intervention. Although participants were instructed to use both tools repeatedly and in real time daily, interviews revealed that the drink-counting app was often used as a daily summary tool, while the breathalyzer was used less frequently − particularly during heavy drinking episodes − as some participants perceived it as shame-inducing (Östh et al., 2023). Therefore, comparisons of the number of drinking days and heavy drinking days based on BrAC likely did not fully reflect actual alcohol use. Furthermore, the lower concordance between BrAC and the other measures supports the interpretation that BrAC should not be used as a stand-alone indicator of alcohol consumption.

Compliance is a known challenge in EMA (Beckjord & Shiffman, 2014), but both tools saw substantial use, with 70 % average daily engagement. Usage was lower early on and declined toward the end, more steeply for the breathalyzer, potentially diluting trends in agreement around week 12, when TLFB recall is most accurate. Still, usage remained generally high, consistent with prior findings of sustained engagement in digital substance use interventions (Nesvåg & McKay, 2018). Given this high utilization, the weak agreement between breathalyzer and TLFB likely reflects low breathalyzer use during drinking occasions. Agreement between TLFB and the other measures was weakest on heavy drinking days. The definitions of heavy drinking vary (Allebeck et al., 2018), and the use of terms like binge drinking, along with their associated cut-offs, has historically been controversial (Pearson et al., 2016, Perkins et al., 2001). In this study, we applied the Swedish definition with an expected BrAC of 0.06 g%, although this threshold is somewhat arbitrary given the lack of universally accepted cut-offs, which are approximate and influenced by multiple factors (Fisher et al., 1987).

Breathalyzer users received thrice-daily prompts, but these may not have aligned with drinking occasions. The weak agreement with TLFB could reflect the 0.06 g% threshold being suboptimal for heavy drinking, and treatment strategies to moderate pace may also have affected BrAC levels. Although TLFB reports exceeded app-based reports, contrary to our hypothesis but consistent with some prior studies (Freeman et al., 2023, Merrill et al., 2020), it remains unclear whether this reflects TLFB overreporting or underreporting in the drink-counting app or breathalyzer. Notably, the drink-counting app better predicted high PEth values, suggesting TLFB may have been inflated in this sample. PEth, a direct biomarker of cumulative intake (Helander & Hansson, 2023), correlated strongly with consumption and drinking days reported by the drink-counting app, consistent with prior studies in clinical and non-clinical populations (Finanger et al., 2024, Helander et al., 2019), supporting the validity of drink-counting app-based reporting. However, reliance on a single PEth test near the 12-week assessment risks misclassification due to inter-individual variation (Helander et al., 2019), and analyses were further limited to participants tested within two weeks of that assessment. The moderate correlation between PEth and drink-counting app-reported heavy drinking days likely reflects inter-individual variability in PEth metabolism (Helander & Hansson, 2023), as well as possible underreporting or underestimation of heavy drinking episodes.

Noteworthy, baseline data indicated a discrepancy between self-reported alcohol use and PEth, suggesting underreporting. With a mean PEth of 0.66 μmol/l—well above the 0.30 μmol/l cut-off—and an average reported past-week consumption of 25 drinks (3.6/day), the strong correlation between PEth and drink-counting app-based reports may help mitigate concerns about underreporting, although analyses were limited to a small sample and a single time point. Considering this, the poor agreement between PEth and breathalyzer reports likely reflect limited use of the breathalyzer during drinking, particularly heavy drinking, reducing its reliability as a consumption measure in this context.

An important consideration when interpreting these findings concerns the timing of the different alcohol measures. TLFB and PEth were retrospective, suitable for pretreatment or follow-up when continuous monitoring is not feasible. In contrast, drink-counts and BrAC were ongoing measures collected during treatment, offering a more dynamic picture of drinking behavior − although participant compliance and reporting accuracy may vary, as observed in this study.

It is also important to note the discrepancy in results between the current study and the previous RCT (Östh et al., 2025). However, in the RCT, effects were observed only during the 26-week follow-up, whereas in this study the focus was limited to the 12-week intervention period. Furthermore, outcomes in the RCT were assessed using TLFB only, while this study relied primarily on app-based data.

### 4.2 Strengths and limitations

The main limitation of this study is its relatively small sample size. Although the original RCT included a larger cohort, only 110 participants were randomized to the drink-counting app or breathalyzer, with further reductions due to complete case analysis for missing data. In this study, drink-counting app and breathalyzer data were compared with retrospective TLFB reports. Although no gold standard exists for assessing alcohol consumption, TLFB was chosen because it is widely used, particularly within addiction treatment settings, and has demonstrated good validity and reliability (Sobell and Sobell, 1992, Sobell et al., 1979).

A key strength of this study is the inclusion of PEth, an alcohol-specific biomarker that reflects recent intake over a few weeks. Unlike self-reports, PEth is highly sensitive and specific to ethanol, capturing cumulative drinking patterns and providing a more reliable measure of sustained use (Helander & Hansson, 2023). Despite the limitation of including a single PEth measure as abovementioned, integrating PEth with self-reported data allows for a more robust evaluation of app-based consumption measures and their clinical utility. A recent study identified ≥ 0.22 μmol/l as the optimal cut-off for hazardous alcohol use (≥120 g/week) in clinical settings (Hammarberg et al., 2024). Including this could have provided more comprehensive comparisons.

Participants were instructed to report their alcohol consumption immediately and regularly using the drink-counting app or breathalyzer. Although these tools were not always used as intended (Östh et al., 2023), they were employed in a real-world clinical setting, thereby enhancing the practical relevance and ecological validity of the study. Nevertheless, our clinical sample consisted of alcohol-dependent individuals with limited psychiatric comorbidity and few social problems, primarily aiming to reduce consumption rather than achieve abstinence. This differs from typical treatment populations (Storbjörk & Room, 2008), limiting the generalizability of our findings to more severe cases.

With the expanding use of mHealth technologies in healthcare, there is growing interest in leveraging digital tools for continuous, real-time monitoring of health behaviors, including alcohol consumption. Our study contributes to this evolving field by evaluating the accuracy and agreement of app-based self-monitoring tools against traditional retrospective assessments and a reliable biological marker (PEth). This provides valuable evidence on the potential and limitations of integrating mHealth applications into routine addiction treatment, informing future development and clinical implementation of such technologies.

### 4.3 Conclusions

The number of drinking days and heavy drinking days reported via the TLFB method were consistently higher than those recorded by the drink-counting app or breathalyzer. Notably, the drink-counting app demonstrated strong agreement with TLFB, further supported by its correlation with the objective alcohol-specific biomarker PEth. In contrast, breathalyzer data generally showed weak agreement with both TLFB and PEth. With the continued expansion of mobile health (mHealth), app-based tools hold increasing potential for integration into clinical practice.

## CRediT authorship contribution statement

**Josefine Östh:** Writing – review & editing, Writing – original draft, Visualization, Investigation, Formal analysis, Data curation. **Andreas Lundin:** Writing – review & editing, Methodology, Formal analysis, Data curation, Conceptualization. **Peter Wennberg:** Writing – review & editing, Methodology, Conceptualization. **Sven Andréasson:** Writing – review & editing, Funding acquisition, Conceptualization. **Anna-Karin Danielsson:** Writing – review & editing, Writing – original draft, Funding acquisition, Formal analysis, Conceptualization.

## Declaration of competing interest

The authors declare that they have no known competing financial interests or personal relationships that could have appeared to influence the work reported in this paper.

## Data Availability

Data will be made available on request.

## Glossary and abbreviations

App: Smartphone application
AUC: Area Under the Curve
BAC: Blood Alcohol Concentration
BrAC: Breath Alcohol Concentration
CCC: Concordance Correlation Coefficient
DD: Drinking Days
EMA: genetic algorithm
Glasklart: Drink-counting self-monitoring app
HDD: Heavy Drinking Days
Heavy drinking: Consuming at least four standard drinks (à 12 g ethanol) on one occasion
iBAC Pro: Mini breathalyser that is connected to a smartphone app via Bluetooth
ICD-10: International Classification of Diseases version 10
mHealth: The use of mobile phones in healthcare
PEth: Phosphatidylethanol 16:0/18:1
RCT: Randomised Controlled Trial
ROC: Receiver Operating Chacteristic
Standard drink: A drink containing 12 g of pure ethanol
TAU: Treatment As Usual
TLFB: Timeline Followback
