# Supplementary material for: Evaluating a drink-counting and a breathalyzer-coupled app for monitoring alcohol use: A comparison with timeline followback and peth biomarker
Source: Addict Behav Rep. 2025 Nov 14;22:100643. doi: 10.1016/j.abrep.2025.100643 (PMC12663845; doi:10.1016/j.abrep.2025.100643)
Supplement: Supplementary Data 1 [file mmc1.docx]

## Supplement A – Intervention timeline

**
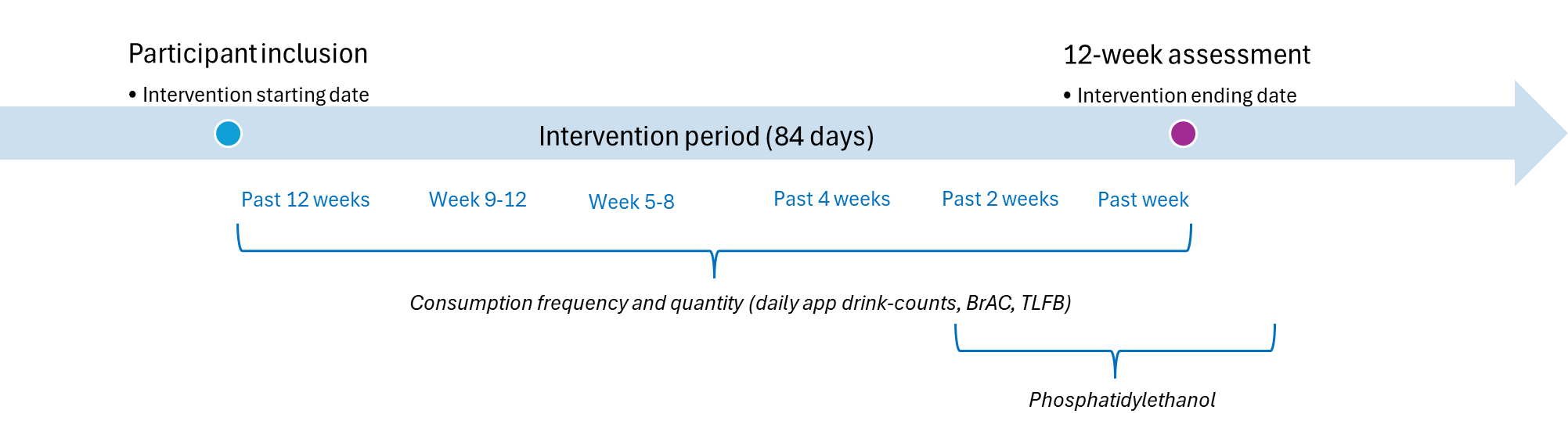
**

Figure A. Timeline of intervention and assessments in the randomised controlled trial.

## Supplement B – Intervention details

### General information and procedures

The drink-counting app, ***Glasklart***, was developed by LifeMesh (<https://lifemesh.se/>) and was freely available for download at Apple App Store (<https://www.apple.com/app-store/>) or Google Play Butik (<https://play.google.com>). After inclusion and randomisation, each participant downloaded the app into their smartphone. Followingly, the app was connected to the online portal by the study nurse by entering a personal identification code that was visible for the participant in the app. Thereafter, the participant could start using the app.

The breathalyser-coupled self-monitoring app, ***iBAC Pro***, was developed by Alcosystems AB (<https://alcosystems.se/>), and the app was freely available for download at Apple App Store (<https://www.apple.com/app-store/>) or Google Play Butik (<https://play.google.com>). After inclusion and randomisation, each participant downloaded the app into their smartphone. To log into the app, the participant provided the study nurse with an email address, who registered it in the online portal, after which the participant got a link to set up a personal account. The external device (i.e., breathalyser) was provided to the participant by the study nurse, usually posted by standard mail, because of the Covid-19 pandemic. After three days, the study nurse made a phone call to make sure the participant had received the device and had been able to connect it to the app to start taking tests.

### Features and theoretical framework

Behaviour change components (BCT’s) describes the “active ingredients” of an intervention aimed at behaviour change and have been motivated to enhance both the reporting of interventions and possibilities of replication (Michie et al., 2013). A set of such components have been developed, addressing aspects of motivation and self-regulation, as well as adjuvant activities and general aspects of communication (Michie et al., 2012). Both apps used in this study, and TAU, included components aiming to enhance motivation and self-regulation, where the specific features of the drink-counting app and breathalyser, with instant, ecologically valid registrations, and outputs, were thought to add additional motivation and engagement, and result in significant reductions in HDD. See Table B.1 for a summary of the BCT’s included in the intervention.

Treatment as usual (TAU) included a manual based on two different, but similar, treatment protocols; Guide to better alcohol habits (GTBA), based on guided self-change, and Guide to controlled drinking (GTKD), based on cognitive behavioural theory (Behavioural Self-Control Training). GTKD usually includes five sessions but was shortened to four sessions to make the two protocols equal in length. In total, 15 clinicians (registered nurses, nurses specialised in psychiatric care, licenced psychologists, or sociologists) employed at the clinic delivered TAU. Clinicians could add discussions and home assignments on moderation strategies, activities, handling of inner triggers, “urge-surfing”, and “maintenance of change”, apart from the above stated procedures. See Table B.2 for an overview of TAU content.

Table B.1 Behaviour Change Techniques of the intervention, based on the
taxonomy by Michie et al (2013).

| **Behaviour Change Technique** | **TAU** | **Drink-counting app** | **Breathalyser** |
| --- | --- | --- | --- |
| Goal setting of behaviour (BCT 1.1) | X | X |  |
| Problem solving (e.g., coping planning and relapse prevention) (BCT 1.2) | X |  |  |
| Action planning (BCT 1.4) | X |  |  |
| Review of behaviour goal(s) (BCT 1.5) | X |  |  |
| Feedback on behaviour (BCT 2.2) | X | X |  |
| Self-monitoring of behaviour (BCT 2.3) | X | X | X |
| Biofeedback (BCT 2.6) | (X) |  | X |
| Social support (emotional) (BCT 3.3) | (X) |  |  |
| Information on antecedents predicting performance of behaviour (BCT 4.2) | X |  |  |
| Information about health consequences of behaviour (BCT 5.1) | X |  |  |
| Monitoring of emotional consequences related to behaviour (BCT 5.4) |  | X | X |
| Prompts/cues (BCT 7.1) |  | X | X |
| Behaviour substitution (BCT 8.2) |  | X |  |
| Social reward (BCT 10.4) |  | X |  |
| Pharmacological support (BCT 11.1) | (X) |  |  |

BCT=behaviour change technique; TAU=treatment as usual; Glasklart=drink-
counting app; iBAC Pro=breathalyser self-monitoring.

Table B.2. Details on content of treatment as usual during the intervention.

|  | **Description of content** | **Home assignments** |
| --- | --- | --- |
| Session 1, week 2 | Feedback on blood samples and questionnaires from initial health check, goal setting of treatment outcome, assessment of motivation, provision of paper alcohol calendar for self-monitoring of standard drinks | Goal setting, alcohol calendar |
| Session 2, week 4 | Revision of goals based on alcohol calendar, risk situations | Risk situations |
| Session 3, week 8 | Goal revision, assessment of risk situations, information on antecedents, coping | Coping plan |
| Session 4, week 12 | Goal revision, initiation of coping plan/plan for maintenance of behaviour |  |
| Overall | Emotional social support (motivational interviewing/cognitive behavioural therapy), feedback on behaviour, information on health consequences. For those with pharmacological treatment, biofeedback on blood samples provided where relevant |  |
| Additional discussions/home assignments | Moderation strategies, activities, handling of inner triggers, “urge-surfing”, and “maintenance of change” |  |

### Training and support

Participants got instructed on how to use the drink-counting app following randomisation. The information was in most cases given orally (video or telephone) because of the Covid-19 pandemic. Participants were also provided with written instructions, including contact details to the study nurse in case they would experience any problems or issues. Participants were instructed to use the drink-counting app for 12 weeks but could potentially continue using it after the intervention period.

Participants got instructed on how to use the breathalyser-coupled app following randomisation. They also got information on different BrAC-levels, that each reminder was active for 60 minutes, and that they needed to wait 15 minutes and rinse their mouth with water before taking a test while consuming alcohol. They were also instructed to try to keep a BrAC-level under 0.06 g% during drinking sessions. The information was in most cases given orally (video or telephone) because of the Covid-19 pandemic. Participants were also provided with written instructions, including contact details to the study nurse in case they would experience any problems or issues. Participants were instructed to use the breathalyser for 12 weeks and had to hand in their breathalyser after the intervention period for it to be cleaned and updated (by Alcosystems), and ready for use by another participant.

### Revisions and updates during the intervention period

#### Drink-counting app

- 2020-12-26: Changes to how data was stored locally on the device.
- Spring 2021: Change of cloud service provider from Amazon Web Services to Elastx due to data security reasons following a new EU-regulation (Shrems II). The move did not affect functionality nor content.
- 2021-06-01: A major update to underlying dependencies, unrelated to user functionality, which may have affected app performance.
- 2021-07-16: Rebuilding of app to squish a crash bug.

#### Breathalyser

- Spring 2021: Change of cloud service provider from Amazon Web Services to Hertz due to data security reasons following a new EU-regulation (Shrems II). The move did not affect functionality nor content.
- 2021-12-03: Available breathalysers stored at the clinic were replaced as they were found to have been outdated and did not function properly.

## Supplement C – Examples of app and portal interfaces

### Glasklart drink-counting app interface (patient perspective):


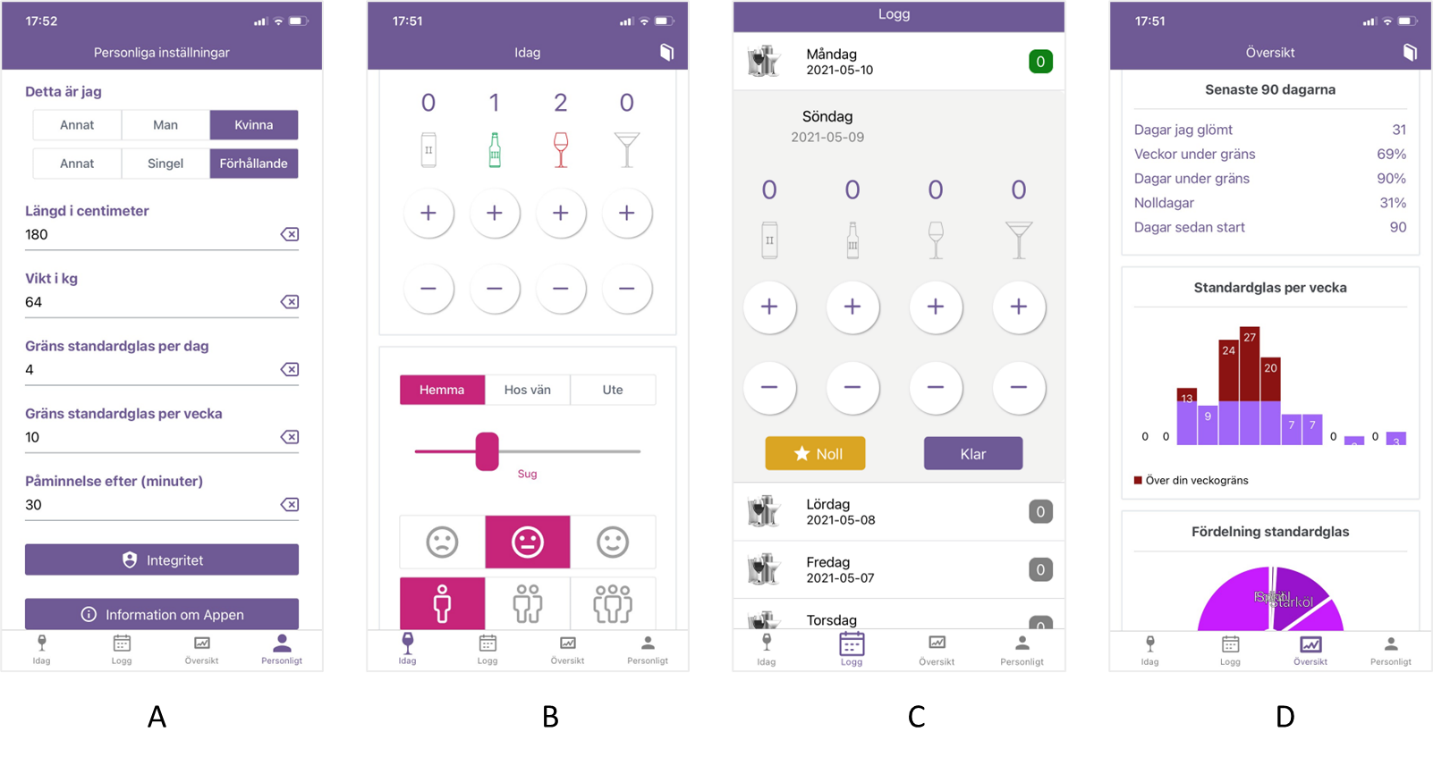


A) “Personal”: Personal details and goal setting (daily and weekly maximum of drinks).
B) “Today”: Daily/instant registration of consumption, location (home/at friend’s/out), craving (scale), mood (sad/neutral/happy), and company (alone/friend/group).
C) “Logg”: Registration of days with zero intake, consumption revision, and retrospective registrations.
D) “Översikt”: Overview of the consumption and additional parameters.

[LifeMesh - Glasklart](https://lifemesh.se/)

### Example of Glasklart portal (below) viewed from the clinician’s perspective:


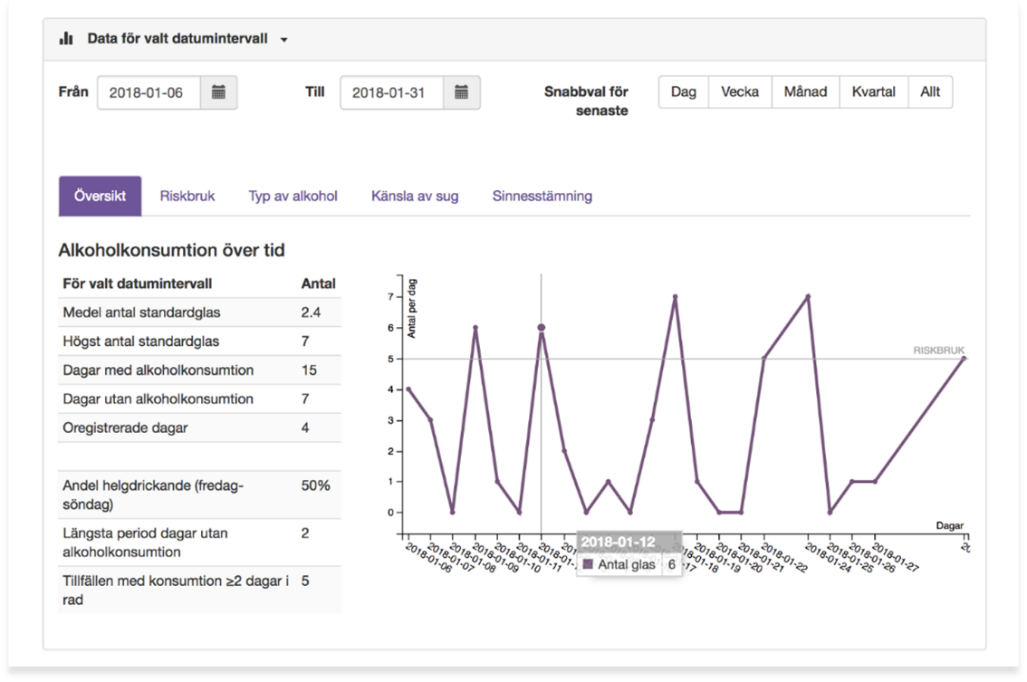


Overview, including registered drinks per day during a chosen time interval, mean and maximum number of drinks, number of days with/without consumption, and proportion of weekend consumption.


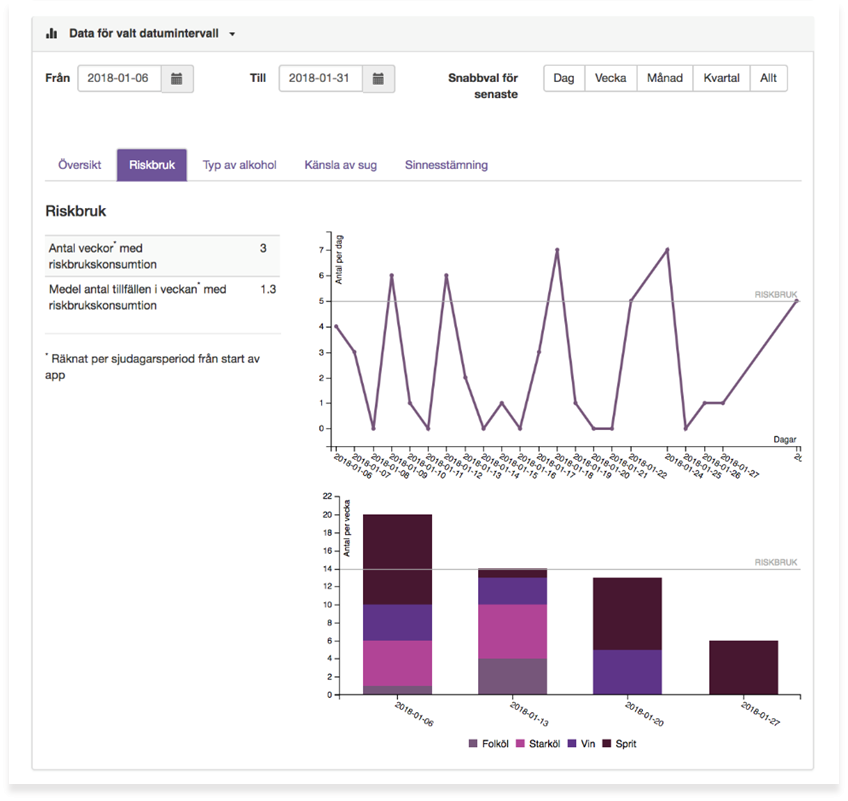


Hazardous drinking, including weeks with hazardous drinking, and mean days per week with hazardous consumption.

### Interface of iBAC Pro app and coupled breathalyser (patient perspective):


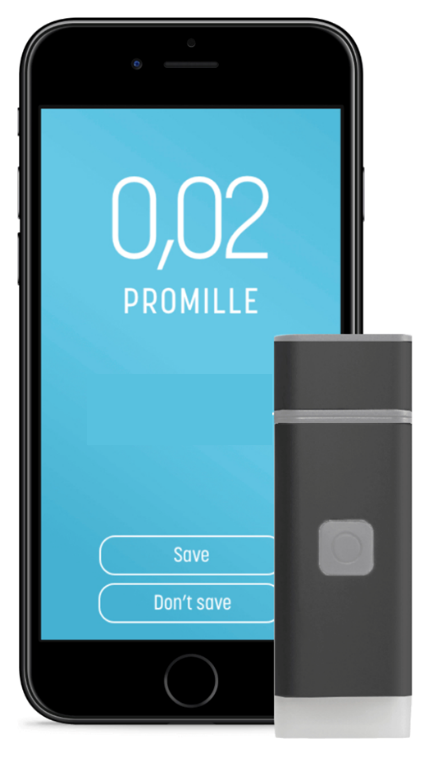


Breathalyser connected to smartphone app by Bluetooth. Picture from: <https://myibac.se/ibac/>


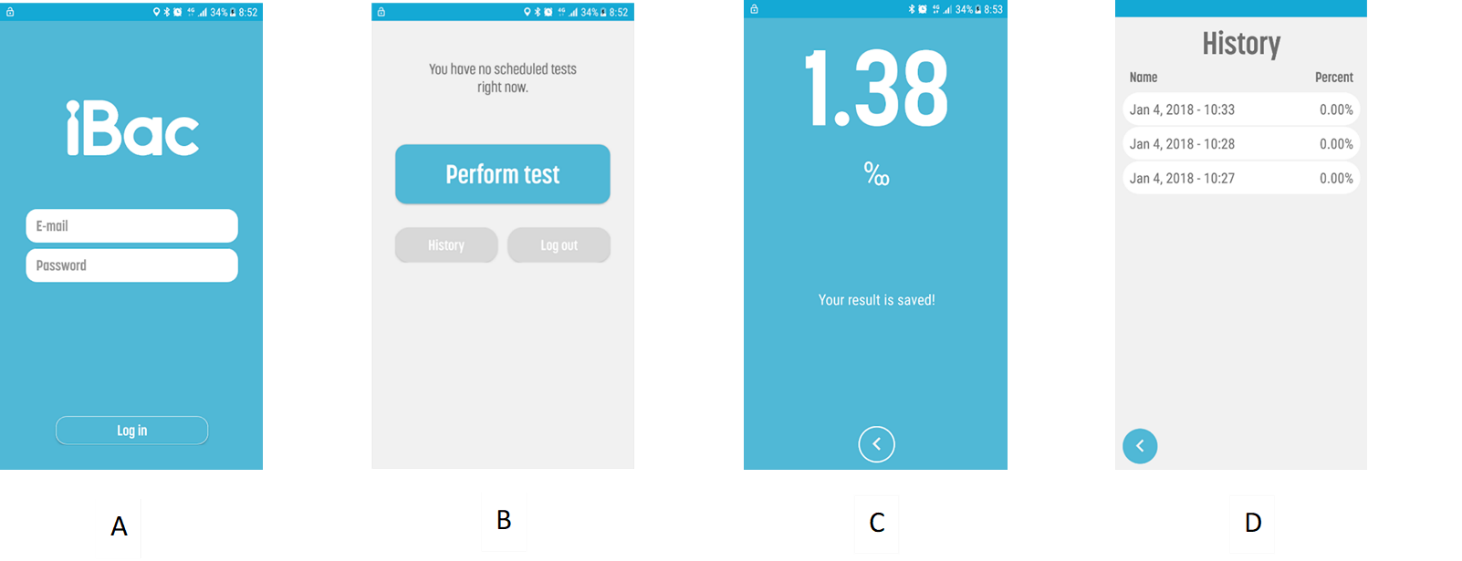


A) Sign-in start page.
B) Performing a test.
C) Instant test result available in the app.
D) History of previous tests.

### Example of iBAC Pro portal (below) viewed from the clinician’s perspective:


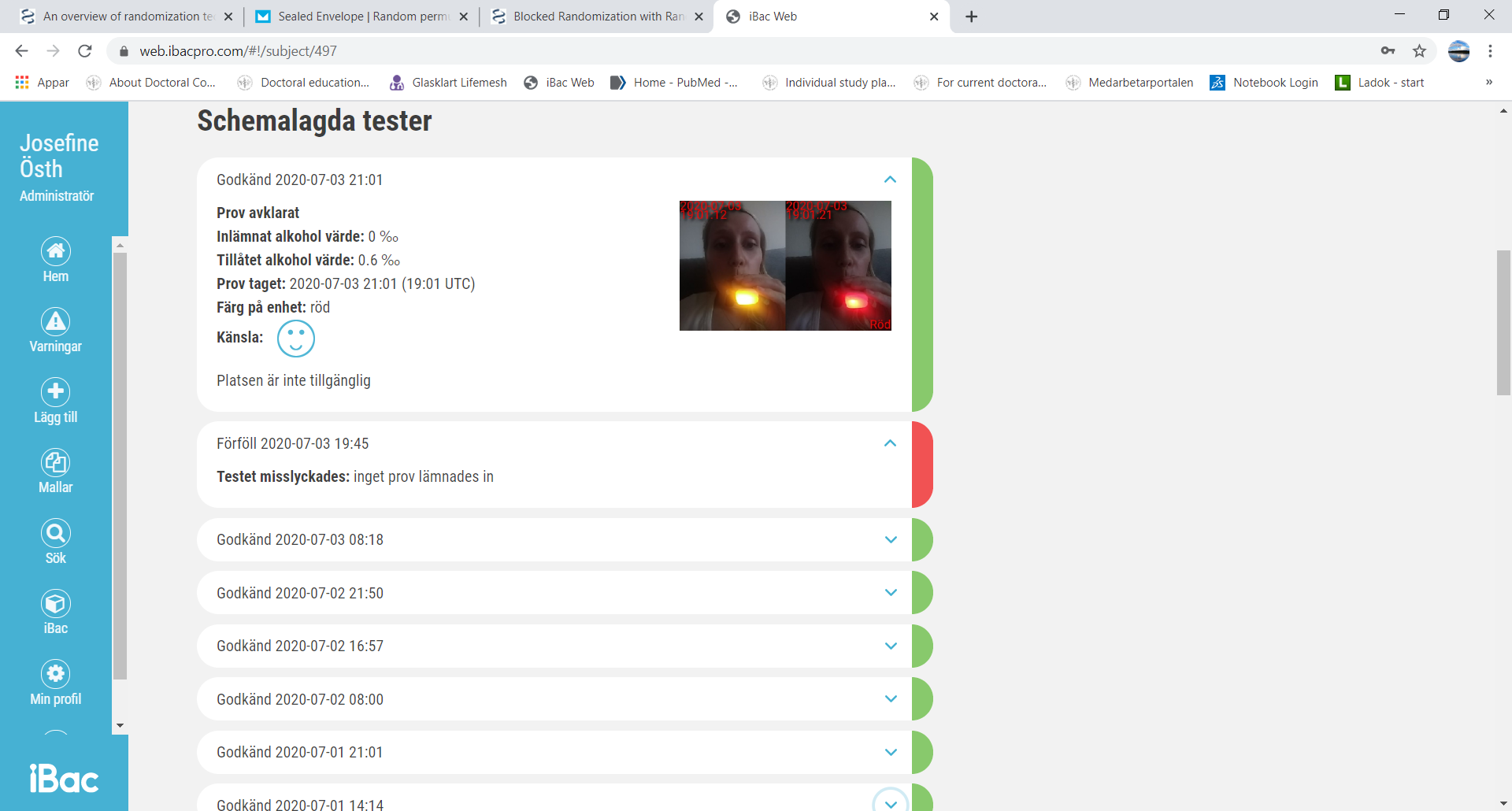


Scheduled and spontaneous breath alcohol concentration tests. Green=performed test with a result under 0.06 g%. Red=non-performed test, or test result >0.06 g%. (NB: this output is from when the main author tested the device, study participants were instructed to re-direct their phone camera if they did not want their picture taken).


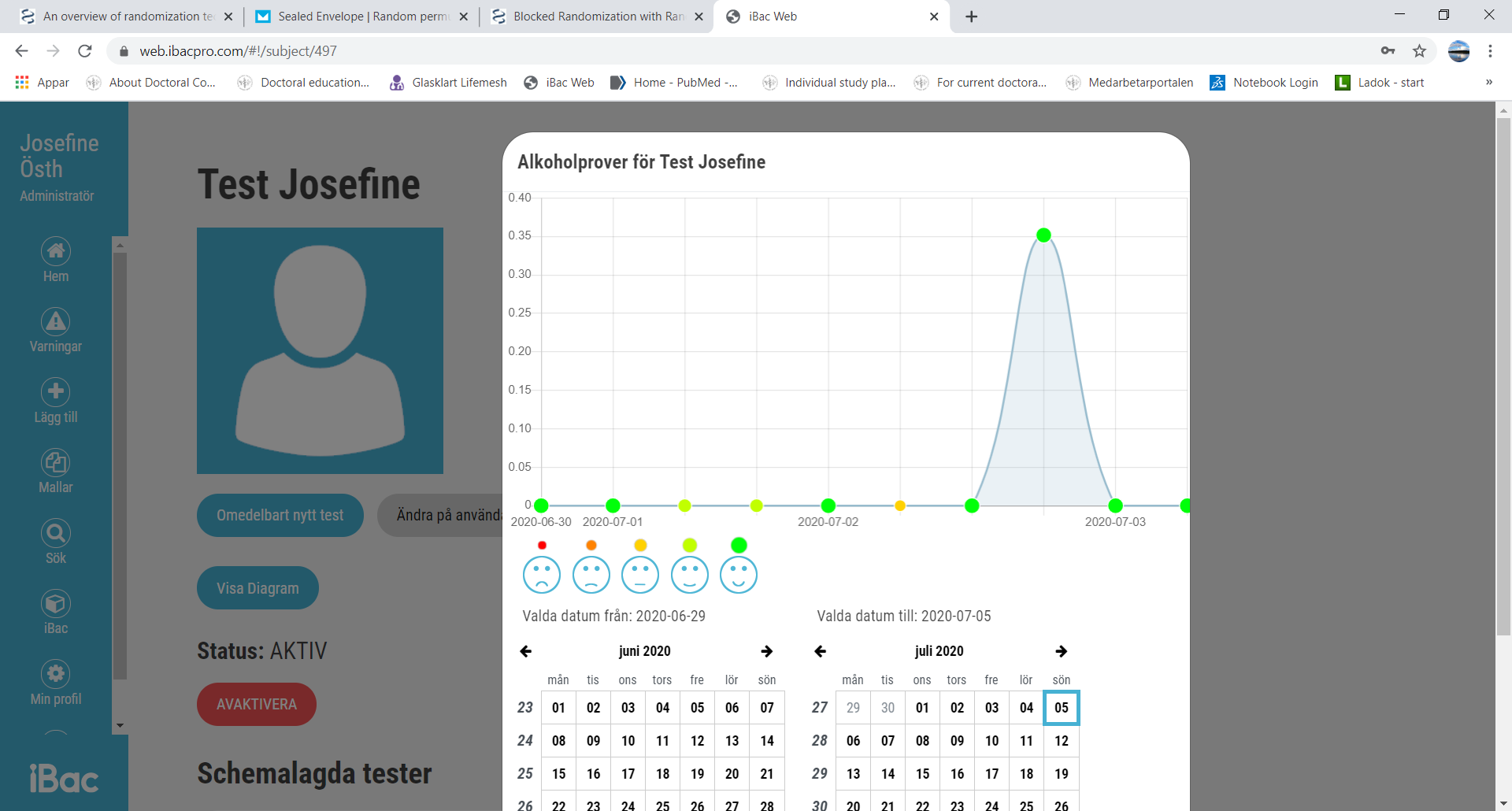


Diagram of daily peak breath alcohol concentration tests, and related mood (colour indicated) during a chosen time interval.

## Supplement D – App users


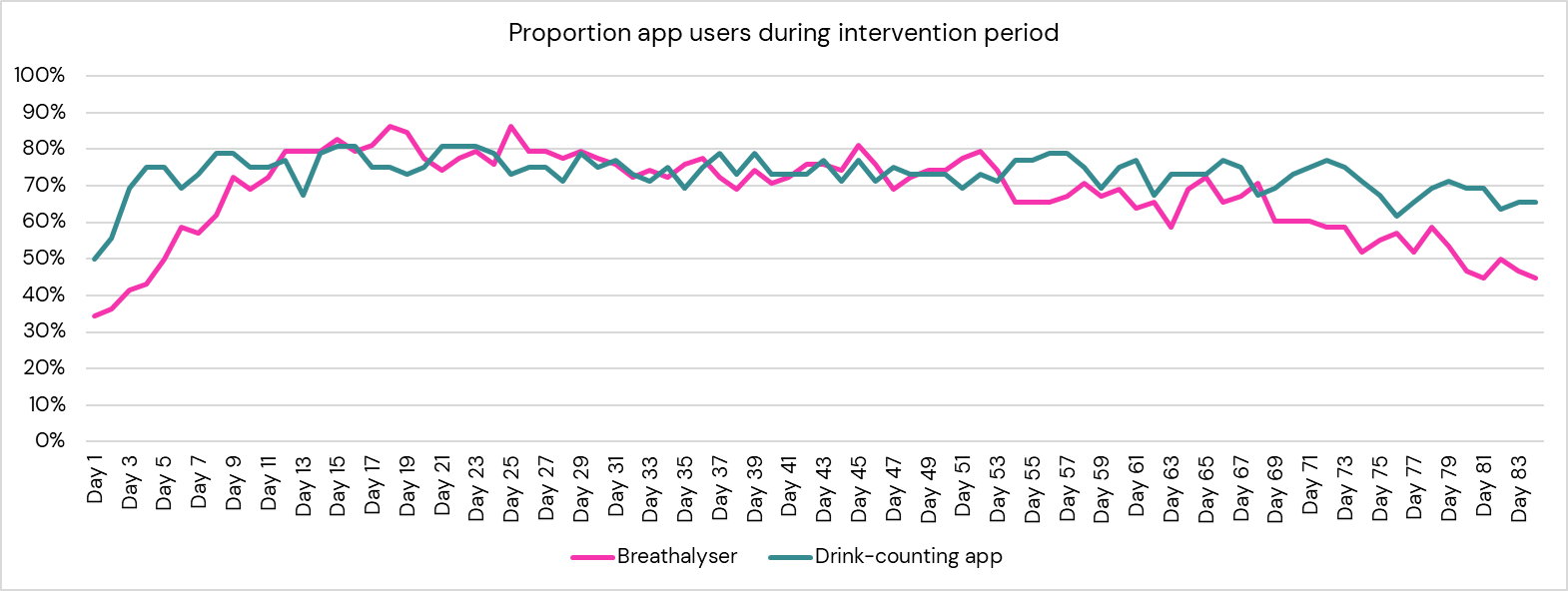


Figure D. Percentage of app users per day during the 84-day intervention.

## References

Michie, S., Richardson, M., Johnston, M., Abraham, C., Francis, J., Hardeman, W.,…Wood, C. E. (2013). The Behavior Change Technique Taxonomy (v1) of 93 Hierarchically Clustered Techniques: Building an International Consensus for the Reporting of Behavior Change Interventions. *Annals of Behavioral Medicine*, *46*(1), 81-95. <https://doi.org/10.1007/s12160-013-9486-6> %J Annals of Behavioral Medicine

Michie, S., Whittington, C., Hamoudi, Z., Zarnani, F., Tober, G., & West, R. (2012). Identification of behaviour change techniques to reduce excessive alcohol consumption. *Addiction (Abingdon, England)*, *107*(8), 1431-1440. <https://doi.org/10.1111/j.1360-0443.2012.03845.x>
